# Insights into the rotational stability of toric intraocular lens implantation: diagnostic approaches, influencing factors and intervention strategies

**DOI:** 10.3389/fmed.2024.1349496

**Published:** 2024-02-13

**Authors:** Xuanqiao Lin, Dongmei Ma, Jin Yang

**Affiliations:** ^1^Eye Institute and Department of Ophthalmology, Eye & ENT Hospital, Fudan University, Shanghai, China; ^2^Key NHC Laboratory of Myopia, Fudan University, and Laboratory of Myopia, Chinese Academy of Medical Sciences, Shanghai, China; ^3^Shanghai Key Laboratory of Visual Impairment and Restoration, Shanghai, China; ^4^Eye Hospital and School of Ophthalmology and Optometry, Wenzhou Medical University, Wenzhou, China

**Keywords:** astigmatism, cataract surgery, toric intraocular lenses, rotational stability, reposition

## Abstract

Toric intraocular lenses (IOLs) have been developed to enhance visual acuity impaired by cataracts and correct corneal astigmatism. However, residual astigmatism caused by postoperative rotation of the toric IOL is an important factor affecting visual quality after implantation. To decrease the rotation of the toric IOL, significant advancements have been made in understanding the characteristics of toric IOL rotation, the factors influencing its postoperative rotation, as well as the development of various measurement techniques and interventions to address this issue. It has been established that factors such as the patient’s preoperative refractive status, biological parameters, surgical techniques, postoperative care, and long-term management significantly impact the rotational stability of the toric IOL. Clinicians should adopt a personalized approach that considers these factors to minimize the risk of toric IOL rotation and ensure optimal outcomes for each patient. This article reviews the influence of various factors on toric IOL rotational stability. It discusses new challenges that may be encountered to reduce and intervene with rotation after toric IOL implantation in the foreseeable future.

## 1 Introduction

According to various studies, it has been estimated that approximately 15 to 29% of patients with cataracts exhibit corneal or refractive-based astigmatism exceeding 1.5 diopters (D) (1, 2). Cataract surgery, which has evolved into a refractive procedure to reduce patients’ dependence on spectacles, can effectively correct regular astigmatism through toric intraocular lens (IOL) implantation (3, 4). However, the rotation of toric IOLs is a significant factor that impacts the visual outcomes (5). Various studies have demonstrated that a misalignment of toric IOLs by approximately 1° can reduce astigmatic correction by approximately 3.3%. Moreover, a misalignment of 30° may fail to correct or even increase astigmatism on a new axis (6, 7). Enhancing the rotational stability of toric IOLs has become a critical focus of contemporary refractive cataract surgery. Recent advancements in technology, particularly in IOL materials and design, have significantly improved the rotational stability of toric IOLs, leading to more precise visual outcomes (8–10).

This article provides a comprehensive review of the research conducted on the rotational stability of toric IOLs, along with the factors affecting their stability (Table 1).

**Table 1:** Risk factors for rotation of toric IOLs in this review.

	Risk factors
Biological parameters	Long axial length
	Large dimensions of the capsular bag
	With-the-rule and oblique astigmatism
Characteristics of toric IOL	C-loop haptic design
	Polymethyl methacrylate materia
	Small toric IOL diameter
Surgery factors	Inaccuracy in marking
	Unsuitable CCC diameter
	Postoperative hypotonia
	Incomplete removal of the OVD
	Grade 4 ACO
	Nd: YAG laser posterior capsulotomy
	CTR was not implanted
Other risk factors	Excessive physical activity

IOL, intraocular lens; CCC, continuous curvilinear capsulorhexis; OVD, ophthalmic viscosurgical device; ACO, anterior capsular opacification; Nd:YAG, Neodymium:YAG; CTR, capsular tension ring.

## 2 Method to evaluate the rotation of toric IOL

### 2.1 Subjective measurement method

Rotation can be directly evaluated using a slit lamp equipped with degree marks on the beam, which is considered the gold standard for assessing the rotation of toric IOLs. By aligning the slit beam with the toric IOL axis markings in the dilated eye, the slit-lamp axis position was recorded and compared with the baseline axis position (initial measurement data) to determine the degree of rotation. This method is simple to perform and has minimal equipment requirements; however, it has certain limitations. As this is a subjective measurement method, the patient’s head position and other subjective factors can influence the results. Previous studies have shown that this method may have an error range of approximately 1–2 (11).

### 2.2 Objective measurement method

With the advancement of technology, various objective measurements have become available in clinical practice to assess the rotation of toric IOLs. Wolffsohn proposed a new photography method called digital retroillumination, independent of eye rotation (12). Using iTrace wavefront aberrometry (Tracey Technologies Corp., Houston, TX, United States), the intraocular astigmatic axis of patients was recorded, and the actual axis of the IOLs inside the eye (perpendicular to the intraocular astigmatism axis) was calculated (13). In the study by Lucisano et al. (14), an anterior segment optical coherence tomography system was used to assess the topographic astigmatic axis and the postoperative position of the axis simultaneously. However, when objective devices, inadequate pupil dilation, fibrosis in later stages and senile rings in the elderly can potentially obstruct the accuracy of toric markings (15). Similarly, the issue of improper patient head positioning may arise Therefore, none of these methods have been able to replace the traditional slit-lamp observation. Deep learning, similar toconvolutional neural network (CNN), may potentially enhance the accuracy of analysis.

## 3 Characteristics of toric IOL rotation

### 3.1 The timing of toric IOL rotation

To ensure the timely detection of early postoperative rotation of toric IOLs and maintain long-term visual stability, it is important to investigate the timing and trends of rotational changes in toric IOLs postoperatively. Rotations of the C-loop haptic IOL were observed as early as 1 h after surgery, with most occurring within the initial 10 days (8, 16, 17). Our team recently reached a similar conclusion in plate-haptic toric IOLs (18). The results revealed that most rotations in the toric IOL occurred within the first 3 days postoperatively, with the maximum degree occurring between 1 h and 1 day after surgery. All the patients included in the study underwent outpatient surgery. This necessitated commuting between the hospital and their homes for postoperative examination the following day, which may have contributed to the maximum rotation of the IOL observed between 1 h and 1 day postoperatively. As for the long-term stability, a previous study showed that the diameter of the capsular bag gradually decreased after cataract surgery. This suggests that IOL stability increased as the capsular bag contracted (19). Seth et al. observed that the rotation change between 1 and 3 months after surgery was significantly smaller than at other time points (20). The rotation of the toric IOL may decrease when the anterior and posterior capsules fuse. Capsule fusion typically begins at approximately 2 weeks, and minimal IOL rotation is observed after 1 month.

### 3.2 The direction of toric IOL rotation

Both clockwise and counterclockwise rotations are possible postoperatively and primarily depend on the IOL design (8, 21). In past studies, the C-loop haptic toric IOL has been described as tending to rotate clockwise (8, 22). However, according to recent research, there is no consensus, as counterclockwise (23) and no direction (24) tendencies have been reported. This can be attributed to a reduction in the circumference of the capsular bag, which may exert pressure on the haptics (25). Regarding the plate-haptic toric IOL, no specific rotation direction has been identified yet (26). Further research is needed to investigate the relationship between the direction of postoperative rotation and the design of toric IOLs.

## 4 Risk factors for rotation of toric IOL

### 4.1 Biological parameters and toric IOL rotation

#### 4.1.1 Axial length (AL) and toric IOL rotation

Early studies suggested that the AL of the eye is one of the main factors contributing to the early postoperative rotation of IOLs following cataract surgery (8, 22, 27). Vass et al. (28) indicated that a longer AL may be associated with a wider capsule width, potentially leading to the implantation of mismatched IOLs. Large capsular bags may reduce friction between the capsular bag and the haptic, decreasing IOL stability (29). Furthermore, in cases where the AL is longer, the implanted IOL tends to be thinner and has a smaller volume, further diminishing the contact and friction between the IOL and capsular bag (27). However, He et al. (30) examined rotational stability in patients with an AL > 25 mm and did not find a significant correlation between AL and rotation. This study also found that in eyes with longer AL, there was no positive correlation between AL and capsule diameter. Moreover, previous research has shown that the plate-haptic toric IOL remains relatively stable and is not significantly affected by an increase in AL (25, 31, 32). For patients with a long AL, the implantation of a toric IOL can still be considered a correction for astigmatism. Nonetheless, factors such as fragile zonules and posterior subcapsular cataracts should be considered in patients with highly myopic eyes. In these eyes, the occurrence of capsular contraction syndrome (CCS) and IOL dislocation is relatively higher, relating to these factors, presenting a significant challenge to the stability of toric IOLs (33, 34).

#### 4.1.2 Dimensions of the capsular bag and toric IOL rotation

Earlier investigations have indicated a strong correlation between rotation and the dimensions of the capsular bag (35). However, currently, there are no devices for the direct and accurate measurement of capsular bag size. Consequently, estimations based on parameters such as the white-to-white (WTW) distance (distance between the horizontal borders of the corneal limbus) and the anterior segment length (distance from the corneal endothelium to the posterior crystalline lens) are sometimes used to approximate the capsular bag dimensions (19, 36). It is recommended to subtract 1 mm from the WTW. If WTW exceeds 12.5 mm, it indicates a significant risk of rotation (37). WTW indirectly reflects the horizontal diameter of the lens capsule. Lens thickness (LT) represents the anterior–posterior diameter of the lens capsule (38). Our study found a strong association among LT, age, and toric IOL rotation. Specifically, our results suggest that toric IOLs may have a higher tendency to rotate in eyes aged ≥70 years and with a LT of ≥4.48 mm (18). With age increasing, there are often increases in LT (39). Li et al. (40) reported a similar finding. Consequently, the combined factors of WTW and LT play a synergistic role in determining the volume of the anterior segment and offer a three-dimensional perspective for estimating the bag size. Utilizing these two parameters in conjunction makes it plausible to anticipate a higher level of predictability in the preoperative assessment of rotational stability in toric IOLs (36). However, some studies have suggested no correlation between LT and toric IOL rotation (17, 30). Additional studies are necessary to confirm the relationship between the LT and rotation.

#### 4.1.3 Direction of corneal astigmatism and toric IOL rotation

The direction of corneal astigmatism may determine the orientation of toric IOL implantation, which could be attributed to the different designs of toric IOLs and the diameter of the vertical capsular bag (41, 42). Several studies comparing loop-haptic toric IOLs found that patients with with-the-rule (WTR) and oblique astigmatism are more prone to significant rotational deviations than patients with against-the-rule astigmatism (43, 44). In addition, in a study conducted by Miyake et al. involving 378 eyes, they reported that out of six eyes with the AcrySof IQ toric IOL that experienced IOL rotation of >20°, all had WTR astigmatism (8). A vertically fixated IOL may exhibit a higher propensity for rotation. Regarding the plate-haptic toric IOL, no definitive conclusion indicates a relationship between the rotation of the toric IOL and the direction of corneal astigmatism. In order to achieve precise analysis and calculation of corneal astigmatism, it is essential to develop novel technologies capable of yielding improved data.

### 4.2 Characteristics of toric IOLs and toric IOL rotation

#### 4.2.1 Shape design and rotation of toric IOL

Toric IOLs can be classified into one- or three-piece designs, with haptic structures available in C-loop or plate haptic configurations. The design of these IOLs is critical for ensuring rotational stability. According to a study by Gyöngyössy et al. (45), one-piece C-loop haptic toric IOLs exhibit excellent long-term rotational stability without postoperative complications.

Kramer et al. compared different brands of C-loop haptic toric IOLs, namely, AcrySof and Tecnis (46). The results revealed that the rotational stability of the AcrySof toric IOL was higher than that of the latter. In a study conducted by Sun et al. (26) the long-term rotational stability of two designs of toric IOLs (AcrySof toric IOL with a C-loop haptic and AT TORBI 709 M IOL with a plate haptic) was comprehensively investigated at the 3-month postoperative mark. The plate-haptic IOL exhibited superior stability compared to the toric IOL with a C-loop haptic (36). The plate-haptic toric IOL was anchored to the capsular bag through its four haptics, resulting in increased friction between the lens and capsular bag, thereby reducing the effects of capsular bag compression and rotation. Additionally, the presence of two positioning holes at the corners of the IOL allowed for the migration of lens epithelial cells, further enhancing the stability of the lens. With its one-piece ‘C’ shaped loop, the IOL might have had a reduced frictional interaction with the capsular bag due to the space between the lens column and the haptic, potentially leading to IOL rotation. Nevertheless, it has also been reported that the loop-haptic design exhibits excellent memory and flexibility, effectively addressing optical fluctuations resulting from capsular bag shrinkage and ensuring stable positioning within the capsular bag (47). The rotational stability of toric IOLs with different shape designs requires further investigation with larger sample sizes.

Therefore, new designs are being constantly developed. A new type of toric IOL, Mini Toric Ready (SIFI S.p.A.), has recently been introduced to the market (48). This innovative design features four fenestrated haptics that enhance the surface area of contact between the IOL and equator of the capsular bag. Fenestrations also facilitate interactions between the anterior and posterior capsules of the eye. As a result, this novel IOL demonstrated superior long-term rotational stability compared to the conventional two-haptic toric IOL. In contrast to the previous generation of TECNIS monofocal IOL, the TECNIS Toric II IOL (Johnson & Johnson Vision) has undergone design modification with frosted haptics. This modification aims to increase the friction between the haptic and equator of the capsular bag, leading to a remarkable improvement in postoperative rotational stability compared to its predecessor (49, 50). We can expect the continuous emergence of new toric IOL designs in the future.

#### 4.2.2 Material design and rotation of toric IOL

The first reported toric IOL made of polymethyl methacrylate (PMMA) exhibited substantial postoperative rotation (2). Various materials, including hydrophobic acrylates, hydrophilic acrylates, and silicone gels, can be used to produce toric IOLs. The materials used in IOLs play a significant role in determining their stability. For example, the adhesiveness of the lens surface is believed to promote stability (51). In particular, hydrophobic acrylic IOLs exhibit stronger adhesion owing to the charge effect and higher fibronectin content (26). Prior research has indicated that toric IOLs made with hydrophobic acrylates exhibit superior postoperative rotational stability compared to those made with hydrophilic acrylates (43, 52). The occurrence rate of postoperative complications, such as rotation, may vary depending on the adhesive force of the posterior capsule for different materials of IOLs.

#### 4.2.3 Size and rotation of toric IOL

Toric IOLs with a smaller overall diameter, especially for eyes with larger capsular bags, can alleviate contact between the lens and capsular bags, thus increasing the risk of rotation. Chang et al. (29) found that a Toric IOL with a total diameter of 11.2 mm (Staar AA4203TL) had a lower incidence of rotation than a Toric IOL with a total diameter of 10.8 mm (Staar AA4203TF). Different IOL sizes may result in variations in surgical duration and technique, potentially leading to rotation.

### 4.3 Surgery factors and toric IOL rotation

#### 4.3.1 Manual marking and computer navigation

Accurate alignment is a fundamental prerequisite for effective refractive correction using toric IOLs. Traditionally, manual marking has been the most frequently described technique for surgical eye marking prior to placing a toric IOL (53). Although the accuracy of manual marking methods is high, computer-guided methods, such as observing plane inconsistencies or tracking dye diffusion, have been developed to overcome the limitations of manual approaches (54). The digital methods are based on previous research, specifically iris fingerprint techniques (55), intraoperative wavefront aberrometry (56), or techniques that use real-time eye tracking based on iris and blood vessel characteristics. Recently, Elhofi et al. (57) found clinically and statistically significant differences between digital and manual marking procedures using Verion, a digital marker published by Alcon Laboratories, Inc. Their findings indicated that preoperative planning and intraocular digital guidance for toric IOL implantation offer advantages over manual marking, resulting in reduced postoperative rotation. Therefore, the Callisto Eye system (Carl Zeiss Meditec AG) is an application of a new virtual reality-based technology that theoretically has the potential to optimize the alignment of artificial IOL and achieve optimal refractive outcomes. It proved that the computer-assisted marker system provided better results than manual marking regarding the postoperative IOL alignment (54). Additionally, using a digital system results in faster intraoperative IOL alignment and shorter overall surgical time (53). In the future, with the involvement of artificial intelligence, we expect improved accuracy and effectiveness of digital marking systems.

#### 4.3.2 Continuous curvilinear capsulorhexis (CCC) and toric IOL rotation

A well-centered CCC that covers approximately 0.5 mm of the IOL is crucial for ensuring the long-term stability of the IOL postoperatively. Insufficient coverage of the IOL owing to excessive tearing of the capsulorhexis causes postoperative rotation. In contrast, a smaller capsulorhexis can lead to anterior capsule contraction, affecting IOL stability. In a study by He et al. (30) there was a positive correlation between TECNIS toric IOL rotation and capsulorhexis size. Therefore, a moderately smaller CCC contributes to improved rotational stability. However, recent studies have attributed the factors affecting IOL rotational stability to the state of anterior capsule coverage over the IOL optical surface, suggesting that complete coverage leads to significantly greater stability than partial coverage. The size of the capsulorhexis did not have a significant impact on rotational stability. Hence, ensuring that the CCC completely covers the IOL optical surface can help achieve postoperative rotational stability without excessively reducing the capsulorhexis size to avoid excessive anterior capsule contraction (17). Considering the commonly used diameter of C-loop toric IOLs in the current market is approximately 6 mm, a capsulorhexis diameter of approximately 5.0–5.5 mm is generally recommended. A retrospective study suggested that the capsulorhexis size is crucial in preventing lens rotation. The study concluded that maintaining a capsulorhexis diameter ranging from 5.0 to 5.8 mm effectively enhanced the rotational stability of Toric IOLs (40). Currently, research on the impact of CCC diameter on the postoperative rotational stability of toric IOLs with plate haptics is limited. Future studies should explore this aspect in greater detail.

#### 4.3.3 Other surgical factors and toric IOL rotation

Studies have shown that postoperative hypotonia and incomplete removal of the ophthalmic viscosurgical device (OVD) can lead to postoperative IOL rotation (58, 59). Postoperative changes in pressure can destabilize the anterior chamber, leading to decreased rotational stability. Tak introduced hydroimplantation, using balanced salt solution (BSS), to maintain anterior chamber shape during the implantation of IOL, instead of using OVD (60). In a study by Chen et al. (61), the hydroimplantation technique yielded outcomes comparable to the conventional technique using an OVD. However, the non-OVD technique offers several advantages, including increased efficiency, reduced surgical time and cost, and elimination of concerns regarding OVD-induced elevated intraocular pressure. Additionally, the hydroimplantation technique ensures complete IOL fixation on the posterior capsule, which is particularly important for AcySof toric IOLs (59). This technique is also beneficial for decreasing the misalignment of toric IOLs during surgery and ensuring postoperative stability. Further research is needed to explore the impact of these operative factors on rotational stability.

### 4.4 Anterior capsular opacification (ACO) grade and toric IOL rotation

The grading of ACO is negatively correlated with the degree of rotation of Toric IOLs and is an independent factor affecting long-term rotational stability (27). One contributing factor is the increased adhesion between the IOL and the capsule caused by anterior capsular fibrosis. In addition, capsular shrinkage resulting from capsular fibrosis can reduce the space available for the IOL, thereby enhancing its stability. As a result, preserving some lens epithelial cells (LECs) from the anterior capsule during cortical aspiration may help minimize rotation and subsequently reduce residual astigmatism (RAS) (62). Therefore, among ACO grades 0 to 3, a higher ACO grade may help reduce Toric IOLs’ rotation (63). However, grade 4 ACO, characterized by excessive capsular bag contraction, can induce anterior folding of the IOL, leading to optical decentration or tilting and severe visual impairment (64). Besides, ACO and sac shrinkage risks with hydrophobic acrylics are lower than those with hydrophilic acrylics and silica gels (65). Nevertheless, the effects of the materials presented here on rotation contradict those of previous reports, which indicated that the hydrophilic acrylic toric IOL exhibited similar rates of postoperative misalignment and surgical repositioning in comparison to the hydrophobic acrylic toric IOL (43). This suggests the necessity for additional research.

### 4.5 Neodymium:YAG (Nd:YAG) laser posterior capsulotomy and toric IOL rotation

Posterior capsule opacification (PCO), a postoperative complication, can directly contribute to the asymmetric contraction of the capsular bag, leading to rotational instability of Toric IOLs (66). However, effective research is lacking to establish a direct correlation between the PCO and lens rotation stability. The most commonly used clinical method to treat PCO, Nd: YAG laser posterior capsulotomy, carries the potential risk of inducing rotational instability in toric IOLs (67). Due to the ACO, preserving a certain number of LECs may help improve the rotational stability of toric IOLs. However, the migration of LECs may also further increase the incidence of PCO. Further research is needed to determine whether anterior capsule polishing, as a method for removing residual LECs following cataract surgery, can enhance the rotational stability of toric IOLs.

### 4.6 Other factors and toric IOL rotation

Excessive physical activity during the early postoperative period is assumed to increase the likelihood of toric IOL rotation, and in a multicenter study conducted by our team, a patient who underwent right-eye toric IOL implantation participated in a marathon race a few days after surgery (68). During the 1-week follow-up, the toric IOL in the patient’s right eye rotated by more than 80°. The patient had undergone toric IOL implantation in the left eye a few weeks prior and remained stable postoperatively. Vigorous activity in the early postoperative period may contribute to toric IOL rotation. As significant rotation of toric IOLs typically occurs within the first 3 days after surgery, it is clinically advisable to advise patients to avoid strenuous activities, especially during the first 3 days postoperatively (68).

## 5 Interventions for toric IOL rotation

### 5.1 Intraoperative interventions for toric IOL rotation

Previous studies have provided evidence supporting using a capsular tension ring (CTR) to limit the rotation of plate-haptic or C-loop haptic toric IOL (11, 69, 70). After the implantation of the CTR, the capsular sac is effectively supported, enhancing its symmetry. The anterior capsules tightened, reducing the contraction of the anterior capsule. In contrast, the posterior capsule closely adheres to the optical portion of the IOL, reducing the asymmetrical contraction of the capsular bag and minimizing the tilt and eccentricity of the IOL, thereby increasing rotational stability. Additionally, by reducing the gap between the posterior capsule and the IOL, the migration and proliferation of lens epithelial cells are prevented, which further contributes to the improved rotational stability of the IOL (71). There is no definitive consensus regarding the ideal timing for CTR implantation. A recent meta-analysis has been conducted by our team (72), and it was demonstrated that the use of CTR significantly improves the rotational stability of toric IOLs by mitigating the influence of LT. Based on these findings, the co-implantation of CTR is strongly recommended for patients with LT of ≥4.5 mm, WTW of ≥11.6 mm, or high astigmatism.

The implantation methods of CTR also exhibited variations. Safran employed a single CTR in conjunction with Toric IOL implantation (73). Sagiv et al. (74) preferred the combined implantation of two CTRs. Ucar et al. (70) employed suture fixation between the CTR and Toric IOL following the combined CTR and Toric IOL implantation. Jiang et al. (75) conducted a 6-month follow-up study on patients to compare the outcomes of two different types of CTR during toric IOL implantation in cataract surgery. One type featured two eyelets; in contrast, the other type had four eyelets. The CTR with four eyelets provided an additional contact area with the toric IOL as it exerted pressure on the posterior capsule. The two additional eyelets helped secure the toric IOL onto the posterior capsule, increasing the contact area and reducing the rotation risk by enhancing friction. However, no consensus exists on whether CTRs should be implanted during surgery. Further research is required to compare the effects of different designs, materials, and surgical techniques on rotational stability.

### 5.2 Postoperative interventions for toric IOL rotation

#### 5.2.1 Surgical approaches for repositioning the toric IOLs

The intraoperative methods for rotating toric IOLs vary depending on the duration since the initial surgery and the extent of attachment between the IOL and the capsular bag (68). If repositioning surgery is performed approximately 3 weeks after cataract surgery, the previous corneal incision is reopened. Subsequently, the OVD is injected into the anterior capsule bag, and the toric IOL is rotated to the desired position. Alignment of the IOL axis is meticulously confirmed, and the incision is hydrated after complete removal of the OVD. If repositioning surgery is performed within 3 weeks postoperatively, two incisions are made on the lateral side of the cornea. One incision is used for anterior-chamber irrigation; the other is used for IOL repositioning. However, this method does not require OVD. Previous studies indicated that repositioning surgery is necessary when the rotation of the toric IOL exceeds 10° from the intended axis. If the rotation is <10° and the change in refractive power of the eye is below 0.50 D, it generally does not significantly impact vision (76).

#### 5.2.2 Timing for the surgery to reposition the toric IOLs

Many other studies have used previously reported timeframes of 1–3 weeks (2, 77). During our team’s retrospective evaluation of 2,745 eyes that underwent toric IOL implantation, a realignment procedure was necessary in 1.68% of cases (68). In this study, the optimal time for repositioning was approximately 15 days, which aligns with previous findings. However, calibrating the lens prematurely may result in lens rotation. Our team found that immediate postoperative repositioning was not the optimal timing choice (68). While delaying the calibration process can result in a more secure fixation of the IOLs within the capsule, it is important to note that rotation occurring after firm fixation has the potential to cause zonular rupture (2, 47, 68). Hence, ensuring proper stability entails selecting the optimal timing for the repositioning procedure and carefully assessing any potential complications in the patient. Research has shown that conducting back-calculation before determining the optimal axis and predicting post-rotational refraction based on the current position and cylinder power of the IOL can lead to improved refractive outcomes, particularly for IOLs with high cylinder power (78). As for recurrent postoperative toric IOL rotation cases, a new technique involving transscleral and trans capsular suture passage through haptics in two opposite directions has been reported (79).

### 5.3 Correction of RAS after toric IOL implantation

In rare cases where a significant RAS cannot be corrected solely by repositioning due to prolonged postoperative time or excessive RAS, various options can be considered, including corneal ablation procedures, arcuate keratotomy, or IOL replacement (58). Among ablative procedures, photorefractive keratectomy and laser-assisted *in situ* keratomileusis (LASIK) yield similar outcomes; however, LASIK is preferred because of its faster visual rehabilitation and satisfactory results (80–82). Excimer LASIK has been proven superior to IOL replacement and piggyback IOL, significantly reducing spherical and cylindrical refractive errors (83). Generally, a waiting period of approximately 3 months after cataract surgery is considered appropriate (84). However, this procedure may not be feasible under certain conditions. For example, it may not be recommended for patients with corneas at risk of developing post-laser ectasia (85). Additionally, the necessary technology may not be readily available in some public health systems and departments. Thus, it is more important to prevent the postoperative rotation of the toric IOL than to focus on correcting it.

## 6 Conclusion

Several factors affect the rotational stability of toric IOLs. Consequently, clinicians should analyze each patient’s situation in a more personalized manner, considering the preoperative refractive status, anatomical structures, surgical techniques, postoperative care, and even long-term management of posterior capsule opacification to minimize postoperative toric IOL rotation to the greatest extent possible. Conventional manual marking has been replaced by image-guided systems and intraoperative aberrometry, which offer markless IOL alignment and contribute to a reduction in postoperative IOL rotation. Whether CTR implantation is necessary to reduce postoperative rotation of toric IOLs remains controversial. With advancements in design and materials, new IOLs are being introduced for commercial use that provide improved visual quality and demonstrate good rotational stability. More relevant studies are anticipated in future research.

## Author contributions

XL: Conceptualization, Investigation, Writing – original draft, Writing – original draft. DM: Conceptualization, Writing – review & editing. JY: Conceptualization, Writing – review & editing.
